# Thermal and non-thermal effects off capacitive-resistive electric transfer application on the Achilles tendon and musculotendinous junction of the gastrocnemius muscle: a cadaveric study

**DOI:** 10.1186/s12891-020-3072-4

**Published:** 2020-01-20

**Authors:** Carlos López-de-Celis, César Hidalgo-García, Albert Pérez-Bellmunt, Pablo Fanlo-Mazas, Vanessa González-Rueda, José Miguel Tricás-Moreno, Sara Ortiz, Jacobo Rodríguez-Sanz

**Affiliations:** 1 Faculty of Medicine and Health Sciences, Universitat Internacional de Catalunya, Barcelona, Spain; 2 Fundació Institut Universitari per a la recerca a l’Atenció Primària de Salut Jordi Gol i Gurina (IDIAPJGol), Barcelona, Spain; 3 Faculty of Health Sciences, Universidad de Zaragoza, C/ Domingo Miral S/N, 50009, Zaragoza, Zaragoza, Spain; 4 Physiotherapy Research Unit, Universidad de Zaragoza, C/ Domingo Miral S/N, 50009, Zaragoza, Zaragoza, Spain

**Keywords:** Achilles tendon, Cadaver, CRet, Musculotendinous junction, Physical therapy

## Abstract

**Background:**

Calf muscle strain and Achilles tendon injuries are common in many sports. For the treatment of muscular and tendinous injuries, one of the newer approaches in sports medicine is capacitive-resistive electric transfer therapy. Our objective was to analyze this in vitro, using invasive temperature measurements in cadaveric specimens.

**Methods:**

A cross-sectional study designed with five fresh frozen cadavers (10 legs) were included in this study. Four interventions (capacitive and resistive modes; low- and high-power) was performed for 5 min each by a diathermy “T-Plus” device. Achilles tendon, musculotendinous junction and superficial temperatures were recorded at 1-min intervals and 5 min after treatment.

**Results:**

With the low-power capacitive protocol, at 5 min, there was a 25.21% increase in superficial temperature, a 17.50% increase in Achilles tendon temperature and an 11.27% increase in musculotendinous junction temperature, with a current flow of 0.039 A ± 0.02.

With the low-power resistive protocol, there was a 1.14% increase in superficial temperature, a 28.13% increase in Achilles tendon temperature and an 11.67% increase in musculotendinous junction temperature at 5 min, with a current flow of 0.063 A ± 0.02. With the high-power capacitive protocol there was an 88.52% increase in superficial temperature, a 53.35% increase in Achilles tendon temperature and a 39.30% increase in musculotendinous junction temperature at 5 min, with a current flow of 0.095 A ± 0.03. With the high-power resistive protocol, there was a 21.34% increase in superficial temperature, a 109.70% increase in Achilles tendon temperature and an 81.49% increase in musculotendinous junction temperature at 5 min, with a current flow of 0.120 A ± 0.03.

**Conclusion:**

The low-power protocols resulted in only a very slight thermal effect at the Achilles tendon and musculotendinous junction, but current flow was observed. The high-power protocols resulted in a greater temperature increase at the Achilles tendon and musculotendinous junction and a greater current flow than the low-power protocols. The high-power resistive protocol gave the greatest increase in Achilles tendon and musculotendinous junction temperature. Capacitive treatments (low- and high-power) achieved a greater increase in superficial temperature.

## Background

Calf muscle strain injuries are common in different activities and sports [1–3].

In different imaging studies there appears to be an injury predominance of the medial head of the gastrocnemius (58 to 65%), the fascial intersection of the medial gastrocnemius and soleus as they merge with the proximal Achilles tendon (66%) [4] and the distal part of the Achilles tendon [5].

Vascular supply has an important effect on tendon tissue repair [6]. Studies in rabbits have shown that when the blood supply in the Achilles tendon is interrupted the tendon fascicles and the tenocytes lost their normal properties, becoming shortened and degenerated and the strands of collagen become acellular and fragmented. Moreover, changes observed in chronic degenerative tendon disorders were shown to be the same as those that occur when the blood supply to the rabbit’s Achilles tendon is disturbed [7, 8]. This demonstrates that vascular supply is one of the key factors in treating tendon tissue.

Capacitive-resistive electric transfer (CRet) therapy is used to treat musculoskeletal injuries [9–12]. CRet is a non-invasive electrothermal therapy classified as deep thermotherapy. It is based on the application of electric currents within the radio frequency range of 300 kHz – 1.2 MHz. This current can generate warming of deep muscle tissues and in turn improve hemoglobin saturation, an increase in deep and superficial blood flow, vasodilation, increase in temperature, elimination of excess fluid and increase in cellular proliferation [13, 14]. Responses such as the increased blood perfusion seem clearly associated with the temperature increase, which is generated due to a physical reaction generated by the flow of current (Joule effect) [15]. The increase in cellular proliferation, however, appears to be associated mainly with the flow of current rather than the temperature increase [16].

CRet therapy provides two different treatment modes: capacitive and resistive. Both treatment modes induce different tissue responses depending on the resistance of the treated tissue. Capacitive mode is provided with an insulating ceramic layer and the energetic transmission generates heat in superficial tissue layers, with a selective action in tissues with low-impedance (water rich). Resistive mode has no insulating ceramic layer, the radiofrequency energy passes directly through the body in the direction of the inactive electrode, generating heat in the deeper and more resistant tissues (with less water content) [17].

The use of deep heating modalities has long been used in the treatment of overuse tendinopathies [18]. It has been reported that the application of heat leads to improved blood flow and oxygen saturation in the Achilles tendon [19, 20]. Thus, thermal agents may be an effective method of treating tendon disorders.

Currently, treatments are based on empirical experience, and the levels of energy and current that must be transferred to produce the desired effects are unknown [9, 11, 13, 21]. There is an article on CRet in Achilles tendinopathies found improved blood circulation of the Achilles tendon but was not able to measure the increase in tendon temperature [22]. Another article studied the changes in temperature with non-invasive devices to monitor deep tissue temperature rather than invasive devices. This was one of the main limitations that the authors themselves commented on in their article [13].

Therefore, conducting a study on cadavers with invasive measurements would help to know the current flow and temperature that deep structures reach in an ethical manner.

Our hypothesis is that the resistive mode is able to have a deeper effect on current flow and generate higher temperature rise in deep structures. The capacitive mode increases more the temperature in superficial structures.

## Methods

### Aim

Our objective was to analyze the thermal behavior and transmission of electric current in the Achilles tendon and the musculotendinous junction of the gastrocnemius muscle with different CRet protocols, using non-living specimens and invasive temperature measurements.

### Study design

This was a cross-sectional study designed to assess the effect of resistive energy/electrical capacitive transfer with the T-Plus Wintecare device on the temperature in the Achilles tendon, musculotendinous junction and superficial region of the calf in cadaveric specimens. The body donor program of the Faculty of Medicine and Health Science of *Universitat Internacional de Catalunya* provided all specimens. The study was conducted in July 2019. The Ethics Committee “Comitè d’Ètica de Recerca (CER) from the Universitat Internacional de Catalunya” approved the study with CBAS201907 reference number.

### Cadaveric specimens

The study material included 5 fresh frozen cadavers: 4 male and 1 female (10 legs). The age range at the time of death was 60–80 years (mean 69.80 ± 6.04). The bodies were stored at 3 °C and brought to room temperature a day before the test to make it stable. The basal superficial, Achilles tendon and musculotendinous junction temperatures were measured prior to any intervention to ensure the same starting values. None of the cadaveric specimens used for this study had evidence of traumatic injuries or surgical scars on the lower limbs.

### Intervention

To better understand the temperature behavior and passage of current in conditions similar to rehabilitative treatments, we applied a power limit similar to that typically applied with a T-Plus device during real-life treatments. This was based on the power level, which is easily identifiable and controllable by the therapist during therapy, and the watts (absorbed power) shown by the device during the therapy.

The power range of a very large T-Plus device ranges from 1 to 300 watts in resistive and from 1 to 450 Volt-Ampere (VA) in capacitive mode.

Two thresholds were identified: *high power* and *low power,* based on the real powers that the therapist typically applies when she/he wants to generate a thermal or non-thermal reaction. On this basis, *high-power* thresholds were set at 90VA in capacitive mode (HPC) and 60 watts in resistive mode (HPR), while low-power thresholds were set at 20VA in capacitive mode (LPC) and 10 watts in resistive mode (LPR). In real-life use, on average, thresholds of 10 watts and 20 VA respect the limit of 0.3 A, while applications at 60 watts and 90 VA are widely-used for a thermal effect.

**Fig. 1:**
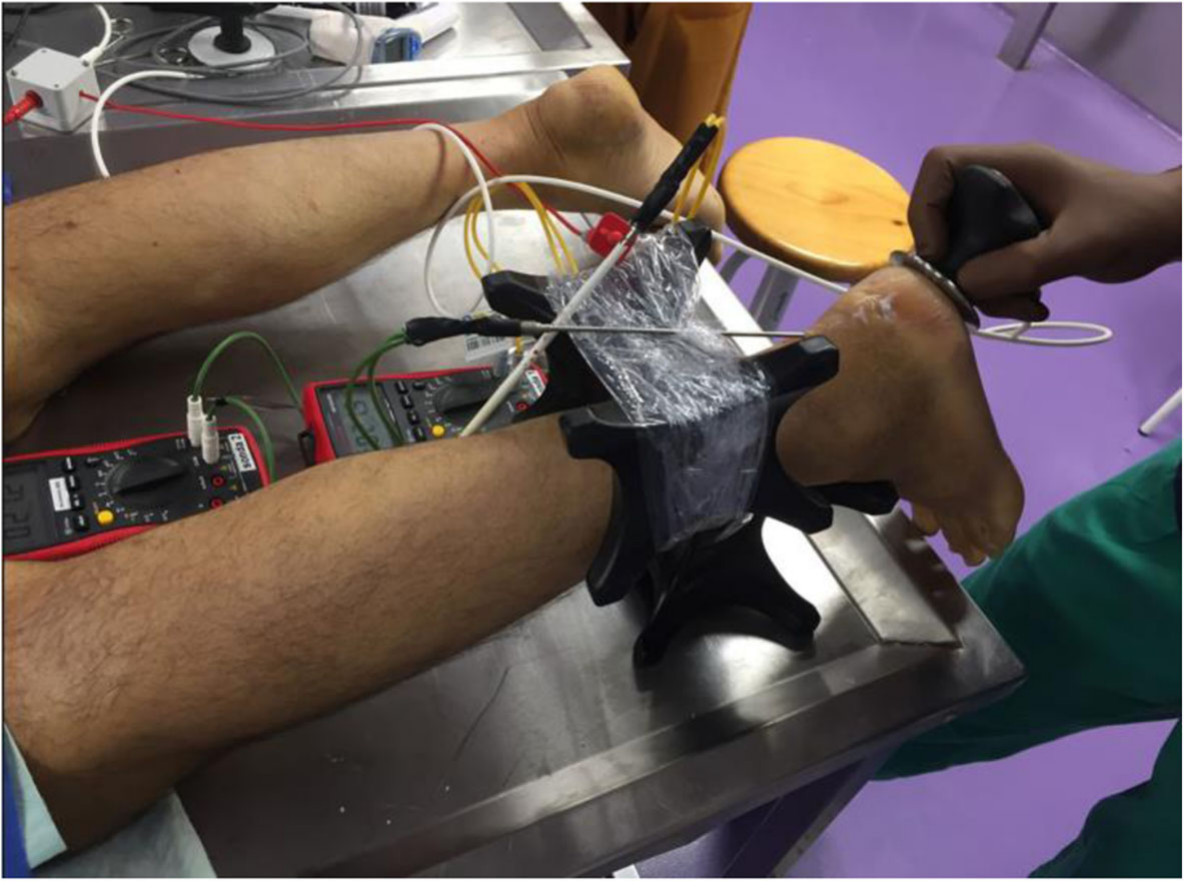
Intervention with T-Plus Wintecare

The 4 interventions (capacitive and resistive mode; low- and high-power) were performed for 5 min each, by a physiotherapist with experience in the use of T-Plus. Dynamic movements similar to those used with real patients were performed with constant pressure to the posterior region of the heel (Fig. 1).

### Experimental procedures

Cadavers were placed in the prone position. Hips were placed in neutral rotation, with the knee in 30° of flexion, and a thermoplastic splint maintained the ankle joint position. The skin was cleaned with chlorhexidine-isopropyl alcohol [23].

The order of the 4 treatment protocols and the specimen (leg side) were both randomized generating a pre-listing through Random.org. by one of the researchers not involved in the recruitment. The temperature generated in the specimen was allowed to return to normal before the next application.

**Fig. 2:**
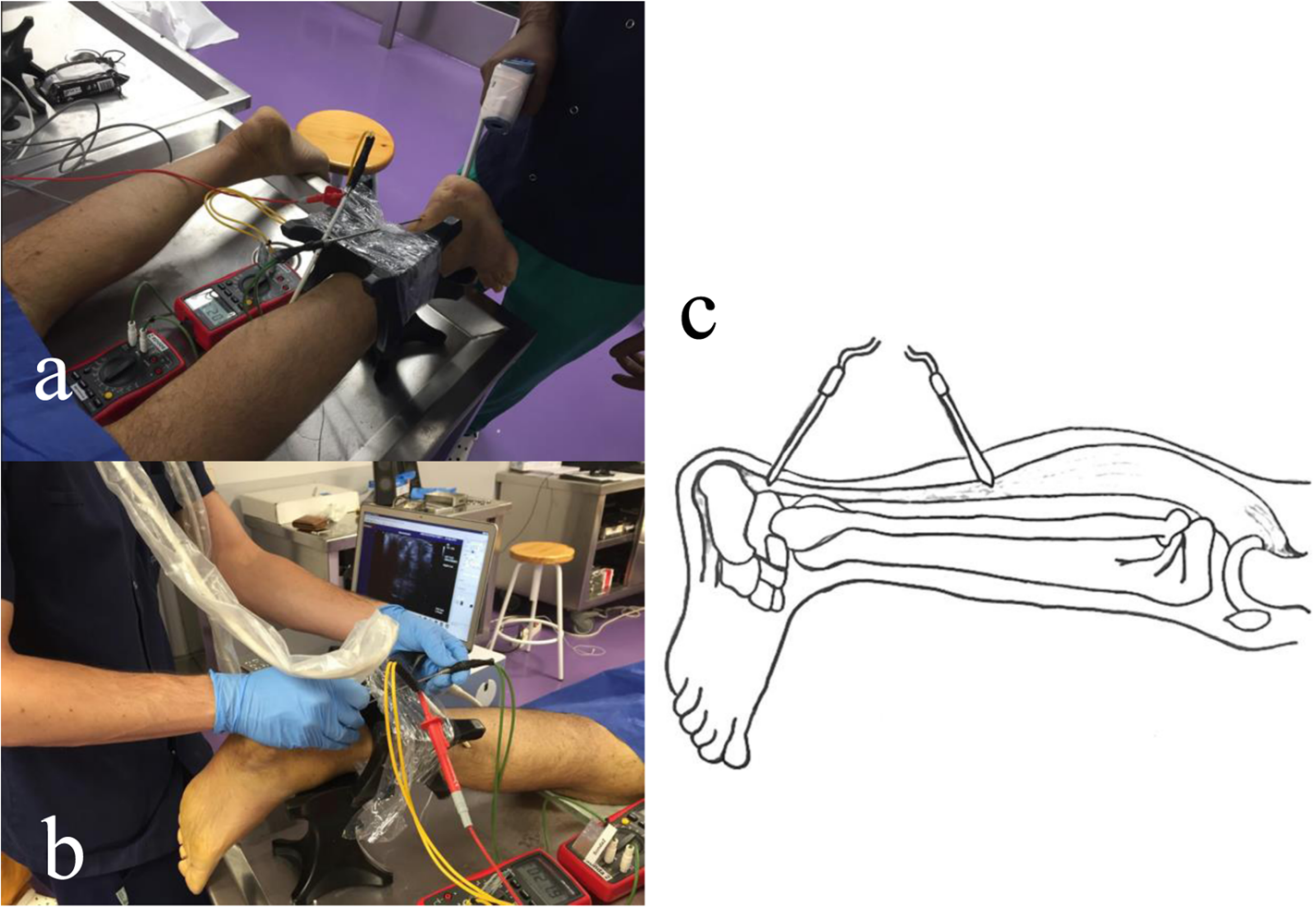
a Temperature measurement with digital thermometer; b Thermocouple placement under ultrasound guidance; c Thermocouple position

All instrumentation received a calibration certificate prior to the study. Thermocouples *“Hart Scientific PT25 5628-15”* were used to measure the musculotendinous junction and Achilles tendon temperature, and a digital thermometer *“Thermocomed”* was used to measure the superficial temperature of the calf (Fig. 2a). Thermocouples were placed under ultrasound guidance *“US Aloka Prosound C3 15.4”*, with a high-frequency linear transducer (USTTL01, 12 L5), by a researcher expert in the use of the instrument (Fig. 2b). One thermocouple was placed in the middle of the Achilles tendon and the other in the musculotendinous junction (Fig. 2c).

The return electrode of the T-Plus was placed on the abdomen of the specimen and the treatment was carried out with the movable electrode of the T-Plus on the heel for 5 min. The initial superficial, Achilles tendon and musculotendinous junction temperatures were measured. These measurements were recorded at 1-min intervals for 5 min and at 5 min after the end of each treatment. Prior to treatment, impedance was always measured (*Multimeter Fluke 8846A)* to ensure that the values marked by the T-Plus Wintecare device were correct. In addition, the current flow of each application was calculated. Using the average voltage divided by the initial impedance.

### Statistical analysis

Analyses were performed using SPSS Statistics version 22.0. Normality of distribution was analyzed using the Shapiro-Wilk test (*p* > 0.05). Mean and standard deviation were calculated for the superficial, Achilles tendon and musculotendinous junction temperatures. The percentages of temperature change respect to baseline temperature were calculated.

For intra-protocol differences, the Friedman test and Wilcoxon signed-rank test were used. Inter-protocol comparisons were performed using the Kruskal-Wallis test and Mann-Whitney U test. A *p* value < 0.05 was considered statistically significant.

## Results

Temperature was recorded at the specified time points during the different protocols. Descriptive outcomes of superficial, Achilles tendon and musculotendinous junction temperature are shown in Table 1. The starting temperature values in the different protocols did not show a statistically significant difference at any of the positions (superficial, *P* < 0.299; Achilles tendon, *P* < 0.396; musculotendinous junction, *P* < 0.871). The current flow in these protocols was stable, with averages of 0.095 A ± 0.03 (HPC); 0.039 A ± 0.02 (LPC); 0.120 A ± 0.03 (HPR) and 0.063 A ± 0.02 (LPR).

**Table 1:** Descriptive outcomes: temperature

		Baseline	1 min	2 min	3 min	4 min	5 min	5 min post-application
Superficial	HPC	21.08 ± 0.68	30.13 ± 4.67	33.69 ± 4.91	35.27 ± 3.62	39.52 ± 4.02	39.63 ± 5.08	28.80 ± 3.02
	LPC	21.38 ± 1.18	24.32 ± 0.93	25.46 ± 1.22	25.72 ± 1.20	26.28 ± 1.26	26.70 ± 0.72	22.30 ± 0.61
	HPR	20.71 ± 1.14	23.63 ± 1.52	24.20 ± 1.10	25.37 ± 1.25	25.02 ± 1.64	26.14 ± 1.90	23.68 ± 0.93
	LPR	21.66 ± 1.25	21.90 ± 1.25	21.97 ± 1.10	21.86 ± 1.20	22.12 ± 1.39	21.88 ± 0.87	20.83 ± 1.00
Achilles tendon	HPC	23.99 ± 1.81	35.45 ± 7.00	35.30 ± 6.40	35.60 ± 6.80	36.33 ± 8.50	36.56 ± 7.79	26.37 ± 1.42
	LPC	23.47 ± 1.67	26.13 ± 2.08	26.52 ± 2.55	26.70 ± 2.55	26.98 ± 2.08	27.58 ± 2.73	25.34 ± 0.88
	HPR	23.97 ± 0.85	47.33 ± 6.65	47.86 ± 5.20	48.57 ± 6.24	49.18 ± 6.32	50.27 ± 6.95	28.11 ± 1.41
	LPR	23.21 ± 1.52	28.33 ± 1.87	28.78 ± 1.79	29.32 ± 1.98	29.60 ± 1.82	29.68 ± 1.87	25.08 ± 0.56
Musculotendinous junction	HPC	19.62 ± 1.98	24.04 ± 4.32	25.45 ± 3.96	25.97 ± 4.19	26.80 ± 4.69	27.33 ± 4.78	22.06 ± 2.33
	LPC	20.03 ± 1.36	21.59 ± 1.85	21.81 ± 1.91	21.99 ± 1.94	22.11 ± 1.91	22.29 ± 1.99	21.06 ± 1.19
	HPR	19.51 ± 1.58	30.30 ± 5.80	32.19 ± 6.09	33.06 ± 6.33	35.29 ± 7.19	35.15 ± 7.24	23.91 ± 2.01
	LPR	20.33 ± 2.62	21.19 ± 1.66	21.57 ± 1.74	21.80 ± 1.76	22.14 ± 1.87	22.42 ± 1.95	20.81 ± 0.93

*HPC* high-power capacitive, *LPC* low-power capacitive, *HPR* high-power resistive, *LPR* low-power resistive.

All protocols showed a progressive increase in temperature at all depths, and a decrease in temperature at 5 min post-application (*P* < 0.001, Friedman test).

The biggest increase in superficial temperature was found at the end of application in the HPC protocol, at 39.63 °C, which represented an 88.5% increase from the starting temperature. However, this temperature decreased in the 5 min post-application to 28.8 °C, representing a 36.9% increase from baseline. The other protocols showed similar values, at ​​around a 26% increase, except for the LPR protocol, which showed almost imperceptible increases of between 1 and 2.3%, registering a 3.8% decrease at 5 min post-application.

**Fig. 3:**
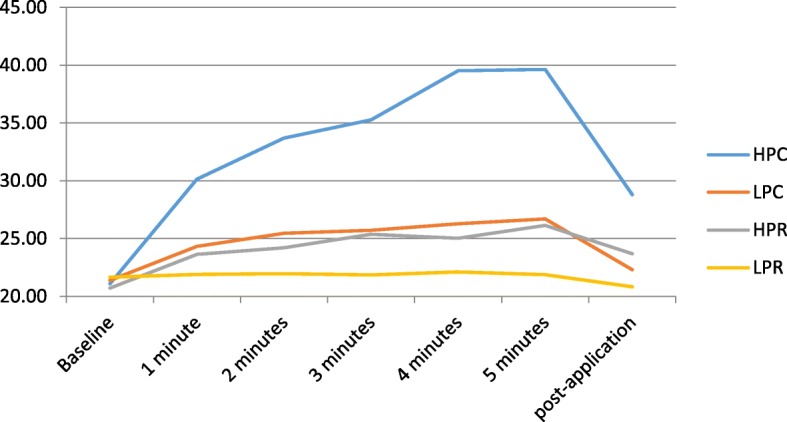
Superficial temperature. HPC: high-power capacitive; LPC: low-power capacitive; HPR: high-power resistive; LPR: low-power resistive

All protocols showed a decrease in temperature at 5 min post-application: the most pronounced decrease was with HPC (Table 1), but this protocol also generated the highest temperature increase (Fig. 3).

Differences between protocols were statistically significant for the difference between baseline and 5 min of intervention and between baseline and 5 min post-application, except for the difference between LPC and LPR for baseline vs 5 min post-application (*P* < 0.853).

**Fig. 4:**
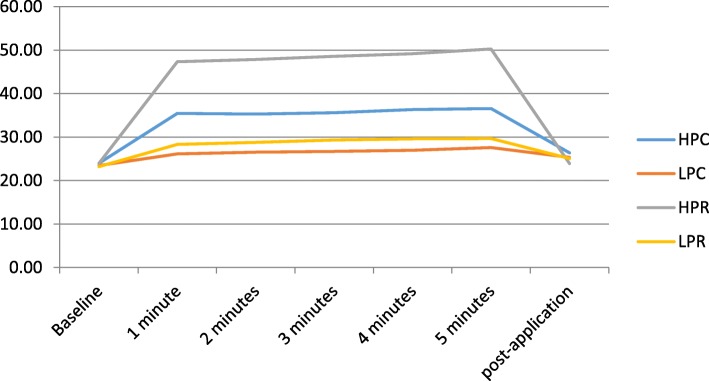
Achilles tendon temperature. HPC: high-power capacitive; LPC: low-power capacitive; HPR: high-power resistive; LPR: low-power resistive

In the Achilles tendon, the HPR protocol produced the biggest temperature increase at 5 min of application, at 50.27 °C, which represented a 109.7% increase from baseline. This value decreased 23.98 °C at the 5-min post-application measurement, representing a 17.4% increase from baseline. In the other protocols, there was less temperature increase, the second highest being 10.3% in the HPC protocol (Fig. 4).

Differences between protocols were statistically significant for between baseline and 5 min of application (*P* < 0.003) except HPC and LPR (*P* < 0.165). Between baseline and 5 min post-application, a statistically significant difference was found between HPR and HPC (*P* < 0.019), LPC and HPR (*P* < 0.002), HPR and LPH (P < 0.002). In the other protocols, no statistically significant difference was reached for baseline vs 5 min of application (*P* > 0.353).

**Fig. 5:**
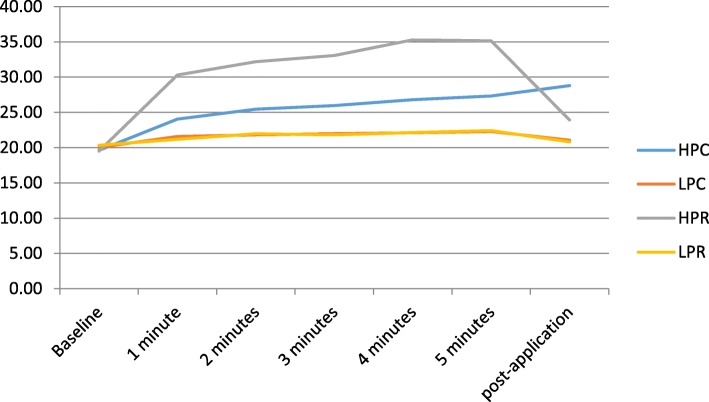
Musculotendinous junction temperature. HPC: high-power capacitive; LPC: low-power capacitive; HPR: high-power resistive; LPR: low-power resistive

Temperatures at the musculotendinous junction reached their highest values at 5 min of the HPR protocol, at 35.15 °C, which represented an 81.5% increase from the starting temperature. The protocol that caused the second-highest temperature increase (by 39.3%) was the HPC protocol, while the rest caused an increase of around 11.5%. However, at 5 min post-application of HPR, temperature decreased to 23.91 °C, which represented a 23.2% increase from baseline, but the HPC increased further, by 1.47 °C, reaching a final temperature of 28.8 °C, representing a 12.6% increase from baseline. In the other protocols, the increase was less than 5.3% (Fig. 5).

The differences between all protocols at the end of the application were statistically significant (*P* < 0.004) except between LPC and LPR (*P* < 0.684). The same was true for the difference at 5 min post-application, which reached statistical significance (*P* < 0.023) except between LPC and LPR (*P* < 0.796).

## Discussion

CRet therapy is one of the methods that has been used in patients with Achilles tendonitis using both the resistive and capacitive modes. Other studies in living subjects have found improved pain levels and increased capillary permeability combining resistive and capacitive mode therapy in healthy male adults [22] and in athletes with different insertional tendonitis [9].

No studies on impedance variation in musculoskeletal tissue between living and cadaveric subjects have been found. It is most likely that the data between living and non-living subjects will vary due to the decrease in blood volume, modifying the resistance values of the tissues. Despite this, there are studies that find differences in impedance in the same subject [24–26] between both extremities [27]. In our study the impedance values in the same subject were similar within the same limb, but varied between limbs. It is likely that the data were slightly different and the temperature would have increased less in the living subjects due to the dispersion generated by the circulatory system.

To our knowledge, this study is the first that evaluates the effects of CRet on temperature and electrical current in deep structures in cadavers. The main findings divided by protocol type are explained below.

### Low-power capacitive

This protocol increases the superficial temperature with a small increase in Achilles tendon and musculotendinous junction temperature. However, despite the small thermal effect, we observed a current flow (0.039 A ± 0.02): this has been associated with cell proliferation in deep structures [14, 16]. This protocol could hypothetically be interesting in acute inflammatory Achilles tendinopathy or acute muscle strain in which it is important to increase cell proliferation [14, 16] and tissue reconstruction without increasing temperature too much [28].

### Low-power resistive

This protocol is similar to the LPC; however, we can see that it has a lower superficial thermal effect, a greater thermal effect at the Achilles tendon and a similar effect at the musculotendinous junction. LPR has a greater current flow (0.063 A ± 0.02) than the LPC, which suggests it may be better at generating cell proliferation [14, 16]. This protocol could be useful in acute inflammatory Achilles tendinopathy or acute muscle strain in which it is important to improve cell proliferation [14, 16] and tissue reconstruction without increasing temperature too much [28]. Previous studies have reported good clinical results with a combination of capacitive and resistive modes [9, 22].

### High-power capacitive

With this protocol, we found an increase in the thermal effect at all depths, especially superficial. In addition, we observed a high current flow (0.095 A ± 0.03), which is associated with a cell proliferation effect [14, 16]. This protocol may be useful in more chronic phases in which the main objective is to improve the viscoelasticity of tissues, especially in chronic tendinopathies or in fibrous scars after sprains such as “tennis leg” [28–30].

### High-power resistive

This protocol achieved the greatest temperature increase at the Achilles tendon and musculotendinous junction. It also registered the highest current flow (0.120 A ± 0.03), which is associated with a cell proliferation effect [14, 16]. This protocol has a greater effect on deeper structures than HPC and could be combined with it to generate further increase in superficial temperature. It could be interesting to combine them if you want to work on chronic superficial and deep pathological structures of the same region [9, 22]. These thermal and current effects may generate mechanical effects on the viscoelastic properties of the structures, which are mainly related with chronic tendinopathies or fibrous scars after sprains [28–30].

### Limitations

As this was a cadaveric study, in which the bodies did not have thermoregulatory blood circulation, it is possible that the effects in living subjects may be minor. It is likely that the living population would not have such a large temperature increase, as circulating blood dissipates heat toward adjacent areas, maintaining the temperature of the treated structures within the desired limits. This process avoids unwanted hyperthermia in nearby tissues, as well as excessive heat during treatment, which can be enough to cause a skin burn [13]. In this type of treatment, patient feedback is important; clearly in this study that was impossible. The temperature increase recorded in this study is probably higher that which would occur in living subjects. In addition, despite being fresh corpses, it is very likely that the muscular properties were not the same as those of living subjects and the average age of the corpses is considerably high compared to the average age of the patients who suffer injuries in this region. However, using body donors allowed us to measure the deep temperature, at the Achilles tendon and musculotendinous junction and make hypotheses about what happens when we apply these treatments in living real patients.

## Conclusion

The low-power treatments had very little thermal effect on the Achilles tendon and musculotendinous junction, but current flow was observed. They may be useful in inflammatory pathologies in which increased temperature is not an objective.

The high-power treatments achieved a greater increase in Achilles tendon and musculotendinous junction temperature, and a greater current flow than the low-power treatments. HPR generated the greatest increase in Achilles tendon and musculotendinous junction temperatures. It may be useful in chronic pathologies in which an increase in deep temperature is desired, to generate viscoelastic changes in the structures.

Capacitive treatments, both low- and high-power, achieve a greater increase in superficial temperature.

More studies are needed in living subjects and other cadaveric studies with an artificial blood system to support these theories.

## Data Availability

The datasets used and/or analysed during the current study are available from the corresponding author on reasonable request.

## Abbreviations

CRet: Capacitive-resistive electric transfer
HPC: High-power capacitive
HPR: High-power resistive
LPC: Low-power capacitive
LPR: Low-power resistive
